# Therapeutic efficacy of external beam radiotherapy combined with anti-PD-L1 inhibition in a preclinical syngeneic head and neck cancer model

**DOI:** 10.1016/j.ctro.2025.101054

**Published:** 2025-10-04

**Authors:** Arshiya Banu, Sophie Langdon, Tanzila Harun, Adam Laouafa, Adam Jones, Kavitha Sunassee, Anthony Kong, Samantha YA Terry

**Affiliations:** a School of Biomedical Engineering and Imaging Sciences, King’s College London, London SE1 7EH, UK; b Faculty of Life Sciences and Medicine, King’s College London, London SE1 9RT, UK

**Keywords:** Head and neck cancer, Immune checkpoint inhibition, Targeted external beam radiotherapy, Radiobiology, Immune cells

## Abstract

Treating high-grade head and neck squamous cell carcinoma (HNSCC) has recently combined immunotherapy with conventional therapies. However, optimizing the scheduling of anti-PD-L1 with external beam radiation therapy (EBRT) requires further research to improve efficacies.

**Methods:**

*In vitro*, MTCQ1, MOCL1, and MOCL2 murine HNSCC cell responses to 2 Gy x 6 X-ray EBRT were assessed in metabolic, clonogenic and γH2AX assays. *Ex vivo*, syngeneic tumors in C57BL/6 mice were stained for haematoxylin and eosin (H&E) and immune cell infiltration. Combination therapeutic *in vivo* studies using MTCQ1 tumors treated with 2 Gy x 6 or 8 Gy x 1 EBRT with sequential and/or concurrent dosing with anti-PD-L1 were also performed.

**Results:**

MTCQ1 cells exhibited the most marked responses to EBRT *in vitro*. H&E analysis revealed highest cellular density and most disperse extracellular matrix in MTCQ1 tumors with infiltration of CD8a+ T cells in tumor centres and macrophages predominantly peripheral. The 2 Gy x 6 EBRT regimen slowed tumor progression; average tumor volumes were 129.2 ± 49.0 mm^3^ on day 10, compared to 234.1 ± 130.7 mm^3^ in the CT-only control (P = 0.039). However, there was also a reduced CD8a+ T cell infiltration on day 3 post complete treatment, with 0.19 ± 0.17 % CD8a+ T cell area compared to 0.91 ± 0.31 % in the CT-only control. Combining EBRT with anti-PD-L1 (delivered either concurrently or sequentially), resulted in greater median survival compared to the CT-only control (33 and 32 days versus 28 days, respectively). Similarly, statistically insignificant improved survival with 8 Gy x 1 EBRT regimen combined with concurrent anti-PD-L1 was observed.

**Conclusion:**

These results reveal spatial distribution of immune cells in the tumor microenvironment and underscore the role of EBRT regimens in modulating immune cell dynamics. This highlights the importance of optimising radiation protocols to inform combination therapy designs in preclinical models.

## Highlights

• The preclinical therapeutic impact of radiotherapy with anti-PD-L1 in head and neck squamous cell carcinoma was studied.
• In vitro, MTCQ1 cells demonstrated significant radiosensitive responses compared to MOCL1 and MOCL2 cells.
• Anti-PD-L1 monotherapy significantly reduced tumor volume, only when treatment was initiated at lower tumor volume.
• Significant changes in CD8a T cells dynamics were observed post 2 Gy x 6 radiotherapy.
• Combining 2 Gy x 6 radiotherapy with sequential anti-PD-L1 treatment led to extended survival by reducing tumor ulceration.

## 1 Introduction

Head and neck squamous cell carcinoma (HNSCC) is the eighth most common cancer in the UK accounting for 12,400 new annual cases [1]. Most HNSCCs originate from the mucosal epithelial layer of oral cavity, pharynx, and larynx. The management is patient- and stage-dependent with most patients with locally advanced HNSCC undergoing primary surgery followed by adjuvant postoperative radiotherapy (+/- concurrent chemotherapy) or primary radiotherapy (+/- concurrent chemotherapy) [[2], [3], [4]]. Traditionally, for curative-intent primary radiotherapy, smaller fractionated doses of 1.8–2.2 Gy are delivered five days per week, with a total dose of 65–74 Gy [5,6]. In the post-operative setting, patients typically receive a cumulative dose of 60–66 Gy in 2 Gy fractions, depending on factors such as surgical microscopic or macroscopic margins with the addition of concurrent chemotherapy for those with positive tumour margins and extra-nodal extension [5,6].

Radiotherapy-treated cancer cells release chemokines, leading to the recruitment of antigen-presenting dendritic cells, which subsequently activate CD4+ and CD8+ T cells thereby facilitating tumor killing [[7], [8], [9]]. Additionally, radiotherapy modulates macrophage polarization, influencing both tissue-resident and tumor-infiltrating macrophages toward either the pro-inflammatory M1 phenotype or the immunosuppressive M2 phenotype [10,11]. Combination treatment with radiotherapy and immunotherapy has demonstrated enhanced antitumor immune responses and improved therapeutic outcomes [12].

Checkpoint inhibitors targeting PD-L1/PD-1 have been characterized as potent activators of T-cell-mediated anti-tumoral immune responses [[13], [14], [15]]. Most recently, PD-1 inhibitors pembrolizumab and nivolumab were approved by the U.S. Food and Drug Administration for treating recurrent and metastatic HNSCC in a first or second line setting after progression post platinum therapy, respectively [16]. Clinical trials have also been carried out to investigate the potential use of anti-PD-1 and anti-PD-L1 inhibitors in combination with chemoradiotherapy. A recent double-blind phase 3 clinical study recruiting 907 locally advanced HNSCC patients showed that concurrent use of anti-PD-L1 avelumab with chemoradiotherapy did not prolong progression-free survival compared to patients who received placebo with chemoradiotherapy (NCT02952586 ) [17]. In another randomised phase 3 study, the addition of pembrolizumab to concurrent chemoradiotherapy also did not improve event-free survival compared to placebo plus chemoradiotherapy in patients with locally advanced HNSCC (NCT03040999) [18]. However, a randomized open-label phase 3 study testing the efficacy of anti-PD-L1 durvalumab given sequentially after chemoradiotherapy in stage III non-small cell lung cancer patients showed increased progression-free survival compared to that in the placebo group (NCT02125461) [19]. Further work is thus still required to determine a regimen of radiotherapy and anti-PD-L1 which will be effective in HNSCC patients.

Here, *in vitro* therapeutic X-ray studies were carried out using the relatively understudied murine HNSCC MTCQ1, MOCL1 and MOCL2 cells. Furthermore, *in vivo* studies were conducted using the syngeneic MTCQ1 tumor model to assess immune responses within the tumor microenvironment following EBRT. Additionally, we evaluated the efficacy of combination therapies using an anti-PD-L1 inhibitors and EBRT administered in different schedules (fractionated doses versus single dose) and timings (concurrent versus sequential dosing).

## 2 Materials and methods

Reagents were purchased from ThermoFisher Scientific (Gillingham, UK), Corning or Sarstedt, unless stated otherwise.

### 2.1 Cell culture

Murine HNSCC MTCQ1, MOCL1, and MOCL2 cells were purchased from Bioresource Collection and Research Center (BCRC) in Taiwan and cultured in Dulbecco’s Modified Eagle Medium with high glucose (1 g/L glucose; Sigma-Aldrich, UK) supplemented with 10 % foetal bovine serum (FBS; Invitrogen, UK), 2 mM L-glutamine, 100 U/mL penicillin and 100 µg/mL streptomycin (Gibco®, USA; complete media) in a humidified 5 % (v/v) CO_2_ atmosphere at 37 °C. Cells were passaged using 0.5 % trypsin containing 0.2 % (w/v) ethylenediaminetetraacetic acid (EDTA; Sigma-Aldrich, UK) followed by neutralization with complete media.

### 2.2 *In vitro* irradiation

External beam X-ray radiation was delivered to cells *in vitro* at 5.63 Gy/min using the SmART + Small Animal Radiotherapy System (Precision X-Ray, Inc., Madison, CT, USA) with an integrated cone-beam CT unit fitted with a 0.3 mm Copper filter.

### 2.3 Viability and metabolic activity

MTCQ1, MOCL1, and MOCL2 cells were plated at 0.1x10^4^ cells/mL in a 12-well plate for AlamarBlue (resazurin) or a 96-well plate for 3-(4,5-dimethylthiazol-2-yl)-5-(3-carboxymethoxyphenyl)-2-(4-sulfophenyl)–2H-tetrazolium (MTS) assays and incubated for 24 h. Cells were left untreated or irradiated at 2 Gy fractions for 6 days (5 consecutive days with a gap of two days followed by 1 more dosing day) with a regular media change. At day 3 post end of treatment, cell viability was monitored using the AlamarBlue assay (ThermoFisher Scientific, UK) and metabolic activity was measured by MTS (Promega, UK) by adding 10 % volume of the respective reagents and incubating cells for 4 h. For details on AlamarBlue analysis, see Supplementary Information. For MTS assay analysis, absorbance was measured at 490 nm, and the percentage metabolic activity was calculated. Absorbances were measured using a SPECTROstar Nano microplate reader (BMG Labtech, Germany).

### 2.4 Clonogenic survival and γ H2AX flow cytometry

Survival fractions were evaluated by clonogenic assays. For γH2AX flow cytometry, cells seeded at 2x10^5^ cells/well in 6-well plates were irradiated at 2 Gy x 6. Flow cytometry was performed to detect γ H2AX on the FACSMelody™ Cell Sorter (BD Biosciences). Data was analysed using FlowJo^TM^ v10.8.1 (BD Biosciences; gating strategy as in Fig. S1). For further details on the clonogenic and flow cytometry studies, see Supplementary Information.

### 2.5 Animal studies and initial tumor growth studies

Animal studies were performed in accordance with the Animals (Scientific Procedures) Act 1986, under Project license and protocols approved local institute Animal Welfare and Ethical Review Body and by UK Home Office. For information regarding housing and humane endpoints, see Supplementary Information.

Given the high prevalence of human papilloma virus-negative HNSCC and its poorer survival outcome in females compared to males, we selected the female mouse model for our studies [20]. For initial tumor growth studies, female C57BL/6 mice (6–7 weeks old, Charles River) were anaesthetized using 2–2.5 % isoflurane in 100 % oxygen. MTCQ1, MOCL1, and MOCL2 cells (1x10^6^ cells/mouse) were resuspended at 1:1 volume in Matrigel. Cell suspension (100 µL) was injected subcutaneously into the right flank of each mouse. Tumor volumes were measured by calliper using V = ½ × (L × W^2^), with “L” being the longest side of the tumor and “W” the longest tumor distance perpendicular to L.

### 2.6 *Ex vivo* sample preparation and histology staining

Details can be found in Supplementary Information.

### 2.7 *In vivo* anti-PD-L1 monotherapy

Female C57BL/6 mice (6–7 weeks, Charles River) were anaesthetized as above, and the right flank was subcutaneously inoculated with MTCQ1 cells at 0.5x10^6^ cells/mouse. Once tumors were 50 ± 15.3 mm^3^, mice were randomized using the Randomice software into groups: (i) control receiving buffer solution (InVivoPure pH 6.5 Dilution Buffer, 2D Scientific, UK), (ii) antibody isotype control at 10 mg/kg (rat IgG2b LTF-2, 2D Scientific, UK), (iii) 5 mg/kg anti-PD-L1, and (iv) 10 mg/kg anti-PD-L1 (rat anti-F3 anti-mouse PD-thL1 (B7- H1) clone 10F.9G2, 2D Scientific, UK). Therapies were injected intraperitoneally twice per week for three weeks, totalling 6 injections.

### 2.8 *In vivo* EBRT − 2 Gy x 6 monotherapy

Female C57BL/6 mice were anaesthetized as above, and the right flank was subcutaneously inoculated with MTCQ1 cells at 1x10^6^ cells/mouse. Once tumors reached 100 ± 32.2 mm^3^, mice were randomized using the Randomice software into CT-only control and irradiated (2 Gy x 6) groups. Mice were first imaged by CT (CT dose of 12 cGy) for treatment planning, and the tumors were then irradiated with 6 fractions of 2 Gy delivered each time across two beams (parallel-opposed beam pair from the anterior-posterior/posterior-anterior directions at 225 kVp, 20 mA) at system optimised angles (Fig. S2). Dose delivery was performed across an average irradiation time of 13.8 ± 0.6 s for beam 1 and 13.8 ± 0.9 s for beam 2. Irradiations did not take place on a weekend. For more information, see Supplementary Information. CT-only control mice underwent CT imaging only. Separately, tumors in irradiated and CT-only control animals were harvested on days 3 and 7 post completion of the irradiation cycle; these were stained for CD8a, CD80, CD206, PD-L1, and Ki67 (see Supplementary Information).

### 2.9 *In vivo* combination therapeutic study

Female C57BL/6 mice were anaesthetized as above and inoculated with MTCQ1 cells at 1x10^6^ cells/mouse. Once tumors reached 100 ± 27.1 mm^3^, mice were randomized using the Randomice software the following groups: (i) CT-only control, (ii) 2 Gy x 6, receiving 6 fractions of 2 Gy irradiation, not at the weekends, (ii) anti-PD-L1, receiving CT imaging and anti-PD-L1 (10 mg/kg) administered twice per week for three weeks, (iv) 2 Gy x 6 concurrent, receiving 2 Gy x 6 irradiation alongside the anti-PD-L1 regimen starting on day 0 of irradiation, and (v) 2 Gy x 6 sequential, receiving 2 Gy x 6 irradiation followed by the anti-PD-L1 regimen starting on day 3 post-irradiation. Anti-PD-L1 was administered intraperitoneally. Irradiations were carried out under anaesthesia as described above. For further detail on study design, see Fig. S3. Mice were allowed to recover and monitored; tumor volumes were also measured as above. Finally, survival was monitored and plotted as a Kaplan-Meier curve.

Using α/β ratios of 10 for HNSCC, the BED and Equivalent Dose in 2 Gy Fractions (EQD2) were measured using the RADformation online calculator; for a single fraction at 8 Gy or alternatively 6 fractions at 2 Gy each, the BED was 14.4 Gy and EQD2 was 12 Gy. A therapeutic study akin to the above was conducted, but combining 8 Gy x 1 EBRT with anti-PD-L1 to evaluate (i) concurrent or (ii) sequential administration, as described in the Supplementary Information and Fig. S4.

### 2.10 Statistics

Results are depicted as average ± standard deviation. Graphs were plotted using GraphPad Prism v10.4.1. Each *in vitro* experiment was performed with technical replicates within three biological repeats. *In vivo* therapy studies used 3–8 mice/group. Statistical significance was calculated using a one- or two-way ANOVA with Tukey’s multiple comparisons test unless stated otherwise. P < 0.05 was considered statistically significant.

## 3 Results

### 3.1 HNSCC *in vitro* cellular responses to 2 Gy x 6 EBRT

**Fig. 1:**
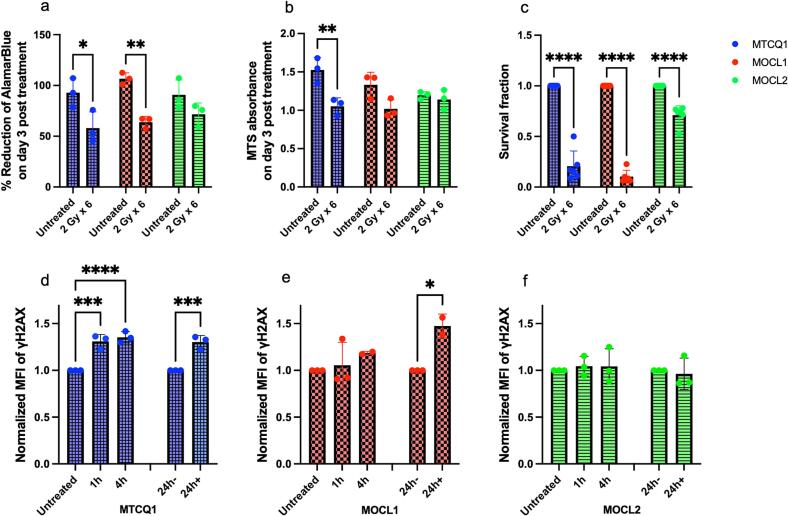
Cell viability, metabolic activity, survival fractions and DNA damage in MTCQ1, MOCL1, and MOCL2 cells treated with or without six fractions of 2 Gy X-rays. (a) Cell viability monitored by AlamarBlue. (b) Cell cytotoxicity monitored by MTS. (c) Colony formation of MTCQ1, MOCL1 and MOCL2 cells analyzed by clonogenic assay. (d, e, and f) Flow cytometry evaluation of γH2AX as a measure of DNA double strand breaks; defined as mean fluorescence intensity (MFI). Values for (c-f) at each time point were normalized to untreated values. Data was analyzed by one-way ANOVA and represented as average ± SD. N = 3. P*<0.05, P**<0.01, P***<0.001, and P****<0.0001.

The therapeutic effectiveness of 2 Gy x 6 treatment was investigated in murine MTCQ1, MOCL1, and MOCL2 HNSCC cells. Through the AlamarBlue assay, it was shown that irradiation of MTCQ1 and MOCL1 cells at 2 Gy x 6 inhibited cell viability at day 3 post the final irradiation dose delivered (Fig. 1a). For example, cell viability significantly decreased from 92.8 ± 14.4 % in untreated MTCQ1 cells to 57.9 ± 16.0 % in MTCQ1 cells that had been irradiated at 2 Gy x 6 (P = 0.032). The viability of MOCL2 cells remained unaffected at day 3 post irradiation (P = 0.14). Cell metabolic viability, evaluated using the MTS assay, showed that irradiation of MTCQ1 and MOCL1 cells with 2 Gy x 6 also inhibited metabolic activity significantly on day 3 post irradiation (Fig. 1b). Compared to respective untreated cells, metabolic viability decreased from 1.5 ± 0.2 to 1.1 ± 0.1 in treated MTCQ1 cells (P < 0.001) and from 1.3 ± 0.2 to 1.0 ± 0.1 in treated MOCL1 cells (P = 0.002). No significant differences were observed in the MOCL2 cells. Compared to respective untreated cells, survival fractions also significantly decreased from 1.0 ± 0.0 to 0.2 ± 0.1, 0.1 ± 0.1, and 0.7 ± 0.1 in irradiated MTCQ1, MOCL1, MOCL2 cells, respectively (Fig. 1c; P < 0.0001 for all cell lines).

Flow cytometry analysis was conducted to assess γH2AX induction. MTCQ1 cells exhibited an increase in the mean fluorescence intensity (MFI) of γH2AX per sample from 1.0 ± 0.0 in untreated cells to 1.3 ± 0.1 as early as 1 h post completion of the 2 Gy x 6 irradiation regimen and remained at the same level up to at least 24 h post irradiation (P = 0.0002; Fig. 1d). In MOCL1 cells, however, a significant increase in γH2AX MFI was observed in irradiated cells only at 24 h post-irradiation (P = 0.0244; Fig. 1e), whereas MOCL2 cells displayed no changes in γH2AX MFI at all (Fig. 1f).

### 3.2 Initial tumor growth studies

**Fig. 2:**
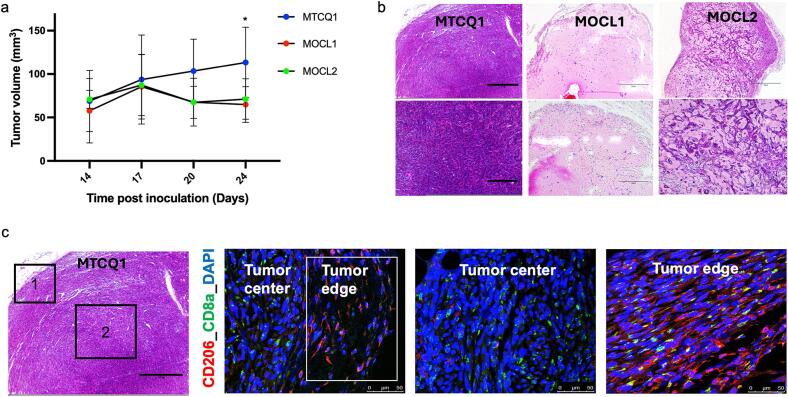
Establishment of subcutaneous murine head and neck models and visualizing the immune niche using MTCQ1, MOCL1, and MOCL2 cells injected subcutaneously into C57B/L6 female mice. (a) Tumor growth as measured by caliper and analyzed by two-way ANOVA. Data is shown as average ± SD, with n = 4 mice/group. *P < 0.05. (b) Representative hematoxylin and eosin (H&E) staining of tumors with scale bar at 750 µm (top panel) and at 300 µm (bottom panel). (c) Representative H&E of MTCQ1 tumor with 1 representing tumor edge, and 2 representing tumor center (with a scale bar at 300 µm), and immunofluorescence confocal images for CD206+ M2 macrophages (red), CD8a+ T cells (green), and nuclei counterstained with DAPI (blue; scale bar at 50 µm).

MTCQ1, MOCL1, and MOCL2 cells were unilaterally inoculated subcutaneously into female C57BL/6 mice and tumor volumes were monitored. At the final timepoint, MTCQ1 tumors exhibited significantly larger volumes compared to MOCL1 and MOCL2 tumors (Fig. 2a). Specifically, MTCQ1 tumors reached an average volume of 113.4 ± 40.5 mm^3^ at day 24 post-inoculation, whereas MOCL1 and MOCL2 tumors measured 64.9 ± 20.5 mm^3^ (P = 0.0117) and 71.3 ± 23.3 mm^3^ (P = 0.0544), respectively. *Ex vivo* H&E staining further revealed that MTCQ1 tumors displayed a highly cellular and compact morphology, with tumor cells arranged in dense sheets and minimal extracellular space, in contrast to the more loosely organized architecture observed in MOCL1 and MOCL2 tumors (Fig. 2b, S5a).

Further immunotyping using confocal microscopy revealed distinct immune cell localization within the MTCQ1 tumor microenvironment (Fig. 2c). CD8a+ T cells were predominantly concentrated in the tumor centre, whereas CD206+ M2 macrophages were localized at the tumor periphery. Staining validation was performed using a full staining procedure on thymus, spleen, and lung sections, alongside secondary-only antibody controls in MTCQ1 tumor tissues (Figs. S5b-c). This confirmed the expected distribution of CD8a+ T cells in the spleen and thymus, while CD206+ M2 macrophages were primarily observed along the epithelial lining of the lung (Fig. S5b).

Histological analysis combined with tumor volume measurements revealed that MTCQ1 tumors established more robustly compared to MOCL1 and MOCL2 tumors. This, along with evidence of increased immune cell infiltration and the clear radiotherapy response *in vitro*, positioned MTCQ1 as the optimal model for future therapeutic investigations.

### 3.3 Therapeutic potential of anti-PD-L1 monotherapy in MTCQ1 tumors

**Fig. 3:**
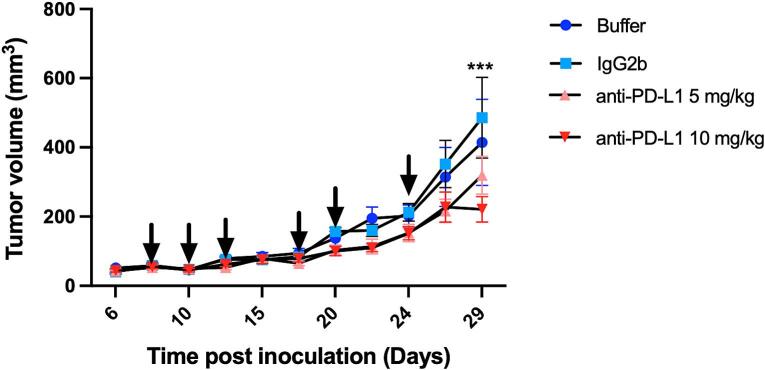
Therapeutic effects of anti-PD-L1 in the MTCQ1 subcutaneous head and neck cancer model. Mice with tumors (50 ± 15.3 mm^3^) were treated with two different doses of anti-PD-L1 antibody (5 mg/kg or 10 mg/kg), IgG2b (10 mg/kg), or with control buffer. Tumor volumes were measured by caliper during the treatment period and analyzed by two-way ANOVA. Data is shown as average ± SD, N = 6 per group. ***P < 0.0001. Arrows refer to timepoints at which mice were injected intraperitoneally with the treatments.

A monotherapy study using anti-PD-L1 at 5 and 10 mg/kg doses was conducted, demonstrating good tolerability as mice maintained stable body weight and exhibited healthy profiles during daily assessments (Fig. S6). Tumor volumes in the antibody isotype control (IgG2b) group showed no significant differences compared to the control buffer group (P > 0.999, Fig. 3). However, by day 29 post-inoculation, the anti-PD-L1 (10 mg/kg) group displayed significantly reduced tumor volumes (220.8 ± 89.7 mm^3^) compared to the IgG2b group (485.6 ± 286.1 mm^3^, P = 0.0003).

### 3.4 Tumor response to 2 Gy x 6 EBRT

**Fig. 4:**
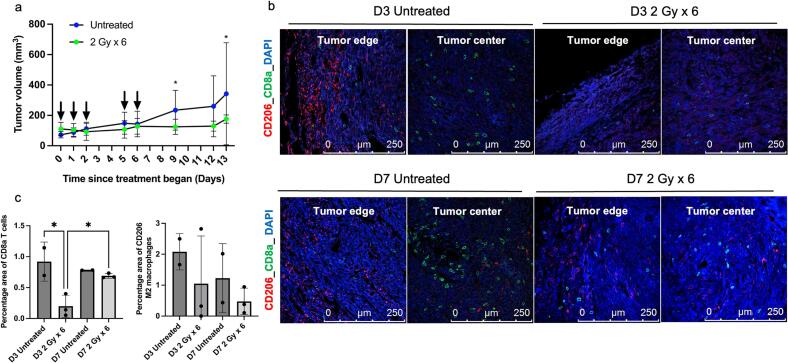
*Ex vivo* tumor analysis: MTCQ1 tumors were irradiated with 2 Gy x 6 regime, and *ex vivo* tumor analysis was performed on day 3 (D3) and day 7 (D7) post treatment. (a) Tumor growth curve analyzed by two-way ANOVA. Arrows (black) indicate the 2 Gy treatment timepoints. (b) Confocal microscopy of MTCQ1 tumors stained for CD8a+ T cells (green), CD206+ M2 macrophages (red), and nuclei counterstained with DAPI (blue). (c) Quantified percentage positive area in (b). Data was analyzed by one-way ANOVA. All the data points are shown as average ± SD, N = 3 mice per group. *P < 0.05.

Mice bearing MTCQ1 tumors were treated with 6 fractions at 2 Gy EBRT (Fig. S2) and tumor growth and the profile of CD8a+ T cells, CD80+ M1 macrophages, and CD206+ M2 macrophages was investigated (Fig. 4, S7). *In vivo* EBRT of tumors at 2 Gy x 6 lowered tumor volumes significantly compared to the volumes of CT-only control MTCQ1 tumors (Fig. 4a). For example, tumor volumes in 2 Gy x 6 EBRT-treated mice measured 129.2 ± 49.0 mm^3^ whereas tumor volumes in CT-only control mice averaged 234.1 ± 130.7 mm^3^ (P = 0.039) at day 10 post treatment.

*Ex vivo* tumor analysis revealed a significant reduction in CD8a+ T cells by day 3 post-EBRT, with the percentage area of CD8a+ T cells decreasing to 0.20 ± 0.17 %, compared to 0.91 ± 0.31 % in the CT-only control (P = 0.0126, Fig. 4b-c). By day 7 post-EBRT, there was a significant recruitment of CD8a+ T cells, with the percentage area increasing to 0.69 ± 0.04 % (P = 0.0425). No significant differences in percentage area of CD206+ M2 or CD80+ M1 macrophages were observed in the 2 Gy x 6 EBRT-treated tumors at any timepoint compared to respective CT-only control tumors (Fig. 4b-c, S7a-b). As shown in Fig. S7c, the 2 Gy x 6 EBRT regimen was well-tolerated and body weight remained stable throughout the treatment period.

Immunohistochemical staining was also performed to assess the expression of PD-L1 and Ki67. While no visible differences were observed in PD-L1 staining, Ki67 expression exhibited notable variations (Fig. S7d). Significant decrease in the percentage area of Ki67 was observed in tumors on day 3 post 2 Gy x 6 EBRT treatment compared to the respective CT-only control tumors (P = 0.0189, Figs. S7d-e).

### 3.5 *In vivo* combination therapy study with 2 Gy x 6 EBRT and anti-PD-L1

Based on the loss of CD8a+ T cells in the MTCQ1 tumor microenvironment on day 3 post 2 Gy x 6 EBRT, therapeutic studies to monitor tumor control and animal survival were designed (Fig. S3). In short, MTCQ1 tumor-bearing animals were administered anti-PD-L1 either concurrently or sequentially with EBRT at 2 Gy x 6. All regimens were well-tolerated (Fig. S8a), and body weight remained stable throughout the treatment period with no adverse effects based on daily health assessment.

**Fig. 5:**
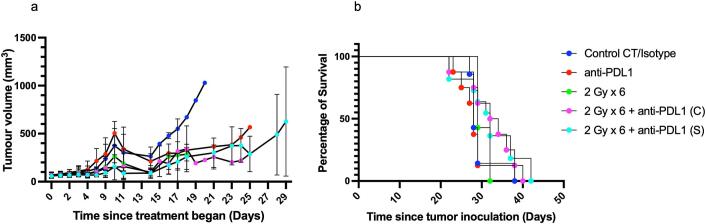
Therapeutic study in female C57BL/6 mice bearing MTCQ1 tumors treated with anti-PD-L1 (10 mg/kg), with radiotherapy at 2 Gy x 6 delivered either individually, concurrently (C) and/or sequentially (S) starting with radiotherapy first. (a) Tumor volumes were measured by calliper from the start of treatment and analyzed by two-way ANOVA, presented as mean ± SD. (b) Kaplan-Meier survival curves depicting cumulative study days for each treatment group. N = 8 mice per group.

Tumor volumes were measured at regular intervals to assess treatment efficacy. On day 11 following treatment initiation, monotherapy with 2 Gy x 6 EBRT resulted in an average tumor volume of 189.0 ± 151.9 mm^3^ compared to the CT-only control 303.1 ± 189.7 mm^3^ (P = 0.0258) (Fig. 5a). Combination therapy with 2 Gy x 6 + anti-PD-L1 showed greater tumor reduction: the concurrent group had an average tumor volume of 158.0 ± 138.9 mm^3^ (P > 0.05 versus CT-only control, 2-way ANOVA across timepoints showed P < 0.0001), while the sequential group showed further reduction with an average tumor volume of 86.4 ± 58.7 mm^3^ (P = 0.019 versus CT-only control). In contrast, monotherapy with anti-PD-L1 did not significantly reduce tumor volumes (338.6 ± 229.7 mm^3^, P = 0.2347, versus CT-only control).

Notably, animals treated with 2 Gy x 6 EBRT monotherapy reached humane endpoints earlier due to early onset of tumor ulceration than those in concurrent or sequential groups. Survival curve analysis using the Log-rank (Mantel-Cox) test showed a significant survival benefit across the treatment groups (P = 0.0279). The median survival was 28 days for both the CT-only control and anti-PD-L1 groups, 29 days for the 2 Gy x 6 group, 33 days for the 2 Gy x 6 + anti-PD-L1 concurrent group, and 32 days for the 2 Gy x 6 + anti-PD-L1 sequential group (P = 0.0279, Fig. 5b).

### 3.6 *In vivo* combination study with 8 Gy x 1 EBRT and anti-PDL1

A single fraction of 8 Gy is biologically equivalent to the fractionated doses of 2 Gy x 6 (∝/β ratio = 10); this single dose was thus also used to evaluate the combination therapy with anti-PD-L1 (Fig. S4). All regimens were well-tolerated (Fig. S8b), and body weight remained stable throughput the treatment period with no adverse effects based on daily health assessment.

**Fig. 6:**
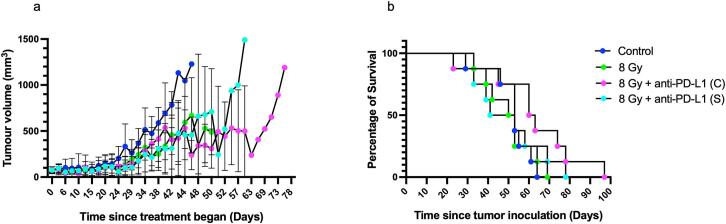
Therapeutic study in female C57BL/6 mice bearing MTCQ1 tumors treated with radiotherapy at 8 Gy x 1 delivered either individually or in combination with anti-PD-L1 (10 mg/kg) given either concurrently (C) or sequentially (S) starting with radiotherapy first. (a) Tumor volumes were measured by calliper from the start of treatment and analyzed by two-way ANOVA, presented as mean ± SD. (b) Kaplan-Meier survival curves depicting cumulative study days for each treatment group. N = 8 mice per group.

Tumor volumes were measured at regular intervals to evaluate treatment efficacy. For example, tumor volumes measured on day 25 post treatment initiation showed that monotherapy with 8 Gy EBRT led to an average tumor volume of 54.8 ± 25.8 mm^3^ compared to the CT-only control 101.6 ± 92.9 mm^3^ (P = 0.2389) although a paired *t*-test across the timepoints showed that 8 Gy irradiation significantly decreased tumor size (P = 0.0005). However, on day 25, combination therapy with 8 Gy + anti-PD-L1 concurrent dosing resulted in a tumor volume of 43.1 ± 16.8 mm^3^ (P = 0.0044 versus CT-only control), whereas the 8 Gy + anti-PD-L1 sequential group showed tumor volumes of 53.4 ± 19.8 mm^3^ (P = 0.6628 versus CT-only control) (Fig. 6a). No significant differences were observed between 8 Gy and 8 Gy + anti-PD-L1 concurrent and 8 Gy or 8 Gy + anti-PD-L1 sequential (P > 0.9999 for both).

Survival curve analysis using the Log-rank (Mantel-Cox) test for trends showed an insignificant survival trend across treatment groups (P = 0.2962; Fig. 6b). The median survival was 53 days for the CT-only control group, 52 days for 8 Gy group, 62 days for the 8 Gy + anti-PD-L1 concurrent group, and 47 days for the 8 Gy + anti-PD-L1 sequential group (P = 0.2962; Fig. 6b).

## 4 Discussion

Combination therapies are being extensively explored to enhance anti-tumor immune cell responses and improve overall therapeutic efficacy [[21], [22], [23]]. Synergy has been observed when combining anti-PD-L1 with radiotherapy in various cancers [24], however, evidence from preclinical models and clinical trials in head and neck cancers has shown reduced or minimal synergistic efficacy, highlighting the need for optimization of treatment strategies [17,25].

Here, we first compared *in vitro* and *in vivo* EBRT and anti-PD-L1 responses and performed *ex vivo* tumor histology for immune cells in irradiated MTCQ1 tumors. *In vitro*, MTCQ1 cells showed consistent therapeutic responses to 2 Gy x 6 EBRT as demonstrated by lowered metabolic activity and survival rates, coupled with significantly higher γH2AX levels, these were not consistently found for MOCL1 and MOCL2 cells. The radiosensitivity observed in murine MTCQ1 cells could potentially be due to reduced DNA double-strand break repair, altered expression of DNA damage signalling and or repair genes, as demonstrated previously in other human HNSCC cells [26].

Furthermore, tumor growth curves and *ex vivo* histology showed significantly higher tumor volumes with high cellular density in MTCQ1 tumors, while more matrix with reduced cell density was observed for MOCL1 and MOCL2 tumors. Together, this indicates the enhanced tumorgenicity of MTCQ1 cells compared to MOCL1 and MOCL2 cells. *In vivo*, MTCQ1 tumors proved responsive to anti-PD-L1 treatment when delivered as a monotherapy, albeit only when anti-PD-L1 treatment was initiated at 50 mm^3^ compared to the treatment initiated at 100 mm^3^ (separate studies; data not shown). Similar findings were observed in a previous study conducted in MTCQ1 models, further supporting the role of tumor size in modulating anti-PD-L1 responsiveness [27]. We further quantified the spatial abundance of CD8a+ T cells, CD80+ M1 and CD206+ M2 macrophages in MTCQ1 tumors, which showed T cells located in the core of tumors and macrophages residing on tumor peripheries. Notably, a dynamic shift in the CD8a + T cells landscape was observed on day 3 post completion of a 2 Gy x 6 EBRT regimen, which could be attributed to direct or indirect radiation-induced CD8a+ T cell depletion. Interestingly, MTCQ1 tumors collected on day 7 post completion of the treatment regimen exhibited significant re-infiltration or recruitment of CD8a+ T cells. This dynamic shift in immune cell abundance underscores the importance of further evaluating different radiation regimens to better understand their impact on immune cell dynamics and functionality, which could inform optimized combination therapies.

The depletion of CD8a+ T cells on day 3 following the 2 Gy x 6 EBRT regimen informed the sequential dosing strategy of anti-PD-L1 in combination therapy. No significant changes in anti-PD-L1 expression were detected post-irradiation, but it was postulated that dampening PD-L1 inhibitory effects on newly recruited CD8a+ T cells would enhance their anti-tumoral response, potentially improving the synergy of combination therapies. Mice that received 2 Gy x 6 or 8 Gy x 1 EBRT exhibited significantly slower tumor progression compared to the CT-only control group, highlighting the therapeutic potentials of these irradiation plans. These radiation doses were chosen as a single fraction of 8 Gy is a commonly used radiotherapy dose in palliative setting and is biologically equivalent to the fractionated doses of 2 Gy x 6 (∝/β ratio = 10). The combination study with 8 Gy x 1 might have reached statistical significance if it had been designed based on immune cell dynamics like the 2 Gy x 6 study. Furthermore, it will be of great interest to test the synergy of EBRT (lower and higher fractionation doses) and anti-PD-L1 in the metastatic model to evaluate abscopal effects of EBRT, and quantify the synergy and changes in the immune population, especially given the fact that exposure to EBRT is limited to a few tumors only.

Alongside other factors, ulceration is listed as a humane endpoint. Interestingly, combining EBRT with anti-PD-L1 treatment, both concurrent and sequential dosing, did not cause significant increase in tumor growth control compared to the relevant irradiation only group; however, combination therapies did result in improved survival by minimising the onset and/or signs of tumor ulcerations. Incurable cancers have ulceration which significantly negatively impact quality of life; for this reason, palliative care focuses on alleviating these associated symptoms [14]. As such, the work carried out here shows that combining EBRT with anti-PD-L1 could potentially enhance patients’ quality of life. However, further validation using patient-derived xenograft models is required to better predict clinical efficacy. In contrast, recent clinical trials evaluating the combination of chemoradiotherapy with anti-PD-L1 reported poorer survival outcomes compared with placebo groups [17,18]. This raises the question of whether chemotherapy may be altering the therapeutic effects of EBRT and anti-PD-L1. In addition, the preclinical study utilized healthy young mice with similar tumor volumes across the groups, whereas the clinical cohort consisted of patients with complex health conditions, higher grade tumors, and diverse dietary backgrounds, which could have further influenced the outcomes.

Impact of tumor size on effective tumor inhibition with anti-PD-L1 suggests that, in HNSCC, therapeutic regimens combining EBRT with anti-PD-L1 might be more effective when initiated at lower tumor volumes. Furthermore, while PD-L1 inhibition has demonstrated therapeutic benefits, it has also led to worsened clinical outcomes in some cases [15], indicating that further investigation is needed to better understand the role of PD-L1 inhibition and the potential negative feedback responses associated with its blockade. Equally, alternatives worth exploring include higher fractionated doses, beyond even 2 Gy x 6. Preclinical studies emphasize the critical importance of selecting an optimal radiotherapy regimen, showing that fractionated protocols such as 2 Gy x 18, and 8 Gy x 3 induce greater immunomodulation and enhance anti-tumor immune responses [[28], [29], [30], [31]]. This effect is particularly pronounced when combined with immunotherapy, including anti-PD-L1, compared to single high-dose radiation, highlighting the potential for synergistic therapeutic strategies [[28], [29], [30], [31]]. Additionally, alternative checkpoint inhibitors, such as those targeting LAG-3 or TIM-3, could be considered for future work.

## 5 Conclusion

This study unveils dynamic, time-dependent shifts in the CD8a+ T cell landscape post-EBRT and highlights the distinct response to radiation of MTCQ1 cells. Notably, the combination therapy demonstrated promising potential to extend overall survival. These findings pave the way for future research to optimize radiotherapy regimens and evaluate their influence on immune infiltration, characterise the immune cells and their respective microenvironment through multiomic platforms to understand the key molecular cues, and thereby establishing preliminary evidence towards enhancing the effectiveness of combination therapies in clinical settings.

## Financial support

AB was supported by Precision X-Ray, Inc. (Madison, CT, USA). AB and the work were also supported by the Immunotherapy and Precision Radiotherapy Fund held by Guy’s Cancer Charity. This work was further supported by the MRC small equipment grant [MR/Y000242/1], MRC project grant [MR/X00841X/1], the Radiation Research Unit at the Cancer Research UK City of London Centre Award [C7893/A28990] and CRUK City of London Centre Radiation Research Centre [RRCOER-Jun24/100002], the Wellcome/EPSRC Centre for Medical Engineering [WT203148/Z/16/Z], and EPSRC programme grant [EP/S032789/1]. SYAT is a member of the NIHR Health Protection Research Unit in Chemical and Radiation Threats and Hazards. For Open Access, the Author has applied a CC BY public copyright license to any Author Accepted Manuscript version arising from this submission.

**Data sharing**.

Research data will be shared upon reasonable request to the corresponding author.

**Disclosures**.

AB was supported in part by Precision X-Ray, Inc. (Madison, CT, USA).

## Declaration of competing interest

The authors declare that they have no known competing financial interests or personal relationships that could have appeared to influence the work reported in this paper.
