# Supplementary material for: Therapeutic efficacy of external beam radiotherapy combined with anti-PD-L1 inhibition in a preclinical syngeneic head and neck cancer model
Source: Clin Transl Radiat Oncol. 2025 Oct 4;56:101054. doi: 10.1016/j.ctro.2025.101054 (PMC12550321; doi:10.1016/j.ctro.2025.101054)
Supplement: Supplementary Data 1 [file mmc1.docx]

**SUPPLEMENTARY MATERIAL**

**SUPPLEMENTARY METHODS**

**AlamarBlue data analysis**

Post treatment and incubation with AlamarBlue, media was transferred onto a 96-well plate to measure absorbance at 570 nm and 600 nm. Percentage reduction of AlamarBlue was calculated using the following equation:

$$Percentage reduction of alamarBlue=\frac{\left[ \left( O2 x A1 \right)-\left( O1 x A2 \right) \right]}{[\left( R1 x N2 \right)-\left( R2 x N1 \right)]} x 100$$

- O1 = molar extinction coefficient (E) of oxidized AlamarBlue (blue) at 570 nm (117216)
- O2 = E of oxidized AlamarBlue at 600 nm (80586)
- R1 = E of reduced AlamarBlue (red) at 570 nm (155677)
- R2 = E of reduced AlamarBlue at 600 nm (14652)
- A1 = absorbance of test wells at 570 nm
- A2 = absorbance of test wells at 600 nm
- N1 = absorbance of negative control well (media plus AlamarBlue but no cells) at 570 nm
- N2 = absorbance of negative control well (media plus AlamarBlue but no cells) at 600 nm

**Clonogenic survival**

Survival fractions were evaluated by clonogenic assays. Cells were seeded at 500 cells/well into 6-well plates and incubated for 24 hours before being left untreated or irradiated with 6 x 2 Gy as above and incubated for 10-12 days. Medium was refreshed every 3 days. Cells were fixed and stained for 20 minutes with 1% crystal violet in methanol (Sigma-Aldrich, Germany). Colonies (≥ 50 cells) were counted, and the survival fraction was calculated considering plating efficiencies of untreated cells.

**γH2AX flow cytometry**

Cells seeded at 2x10^5^ cells/well in 6-well plates and incubated for 24 hours were irradiated at 2 Gy x 6 and cell suspensions were collected at 1, 4 and 24 hours post the last irradiation. Cells were then fixed in 4% paraformaldehyde for 10 minutes at room temperature, washed with PBS, permeabilized with 0.1% triton-X-100 (Sigma-Aldrich, Germany) in PBS for 10 minutes on ice, and resuspended in blocking buffer (1% BSA, 0.2% Tween-20 in PBS) for 30 minutes at room temperature. Cells were subsequently centrifuged for 5 minutes at 500 x *g* and incubated in blocking buffer containing rabbit anti- γH2AX (1:250 dilution, Cell Signaling Technology, US) overnight at 4 °C. Cells were washed in PBS with 0.01% Tween-20 (PBS-T) before incubating for an hour at room temperature with 1:100 goat anti-rabbit IgG Alexa Fluor™-488 (ThermoFisher Scientific, UK) in blocking buffer. Cells were washed with PBS-T and resuspended in 2% BSA and 2 mM EDTA in PBS. Flow cytometry was performed to detect γH2AX on the FACSMelody™ Cell Sorter (BD Biosciences). Data was analysed using FlowJo^TM^ v10.8.1 (BD Biosciences; gating strategy as in Figure S1).

**Animal housing and humane endpoints**

Mice were maintained within the KCL Biological Services Unit under specific pathogen-free conditions in a dedicated and licensed air-conditioned animal room (23 ± 2°C and 40 - 60% relative humidity) under light/dark cycles lasting 12 hours/day. They were kept in individually ventilated standard plastic cages (IVC; 501 cm^2^ floor space; from Tecniplast) including environmental enrichment and bedding material in the form of sterilized wood chips, paper stripes and one cardboard roll per cage. Maximum cage occupancy was four animals, and animals were moved to fresh cages with fresh environmental enrichment and bedding material twice per week. Sterilized tap water and food were available ad libitum; food was PicoLab Rodent Diet 20 (LabDiet) in the form of 2.5 x 1.6 x 1.0 cm oval pellets that were supplied at the top of the cages.

Studies were terminated when mice reached endpoints of the study or humane endpoints. Humane endpoints were determined based on signs of animal discomfort or pain, significant weight loss (20% weight loss in the absence of other clinical signs or 15% weight loss in the presence of other clinical signs), when tumor volumetric size reached 1500 mm^3^ or if progressed towards likely ulceration, example skin surface of tumor starting to thin or appear red. Mice were culled via cervical dislocation with secondary death confirmation by the cutting the artery at the thigh region.

***Ex vivo* sample preparation**

Extracted thymus, spleen, lungs, and tumors were fixed in 4% paraformaldehyde in PBS overnight at 4°C, processed through alcohol series and xylene, and paraffin embedded. Sections (5 μm) were cut and mounted on SuperFrost slides (ThermoFisher Scientific, UK) for further evaluation.

***Ex vivo* immunofluorescence**

Paraffin-embedded tissue sections were dewaxed in xylene and rehydrated through a decreasing series of ethanol concentrations to water. Sections were next subjected to heat-induced antigen retrieval in citrate buffer. Sections were blocked in PBS-1% BSA (blocking buffer) for 30 minutes, washed with PBS-T, and incubated overnight with monoclonal rat anti-CD8a (1:250) (Invitrogen, UK), polyclonal rabbit mannose receptor anti-CD206 (1:300) (Abcam, UK) or polyclonal rabbit anti-CD80 (1:250) (Abcam, UK) in blocking buffer, in a humidified chamber at 4°C. Sections were subsequently washed in PBS-T and incubated with respective polyclonal goat anti-rat AlexaFluor-488 (Invitrogen, UK) or anti-rabbit AlexaFluor-555 (Abcam, UK) at 1:200 dilution in blocking buffer for an hour at room temperature in the dark. Sections were washed and mounted with media containing DAPI (Abcam, UK). Slides were scanned and images captured on Leica confocal microscope and processed in Fiji (version 2.14.0). Percentage staining area was obtained by thresholding and was normalized against total area of DAPI.

***Ex vivo* immunohistochemistry for haematoxylin and eosin, PD-L1 and Ki67**

Paraffin-embedded tumor sections were stained with haematoxylin and eosin (H&E).

Separately, after processing and antigen retrieval steps, sections were washed with distilled water and then PBS before Avidin/Biotin Blocking Kit (2B Scientific, UK) was applied. Sections were washed and then blocked with 10% normal rabbit serum for 30 minutes at room temperature. The sections were washed and incubated with the polyclonal rabbit anti-PD-L1 (R&D Systems, UK) 1:50 dilution in PBS-1% BSA (blocking buffer). The next day, sections were washed 3 times with PBS and incubated with secondary biotinylated anti-rabbit antibody diluted 1:400 in blocking buffer for 30 minutes at room temperature in the dark. Next, VECTASTAIN® Elite® ABC-HRP Kit (Vector Laboratory, UK) was applied to sections and incubated for 30 minutes in the dark at room temperature. Finally, 3,3′-diaminobenzidine (DAB) solution (Universal Biologics, UK) was applied to slides for 8 minutes at room temperature.

For Ki67 staining, the sections after antigen retrieval were washed in PBS and were incubated in blocking buffer for 30 minutes at room temperature. The sections were incubated with monoclonal rat anti-Ki67 (eBioscience, UK) 1:100 dilution in blocking buffer overnight in a humidified chamber at 4 °C. Next day, sections were washed with PBST and incubated with 3% H_2_O_2_ solution for 15 minutes and rinsed thoroughly in tap water. Sections were incubated with secondary HRP-conjugated anti-rat antibody for an hour at room temperature. The sections were washed with PBST and then developed using the peroxidase substrate DAB kit (Vector labs, UK).

All slides were counter stained with haematoxylin and mounted with DPX and scanned (Hamamatsu Nanozoomer S360 scanners). Images were processed using NDP.view2, and Ki67 staining was quantified in Fiji (version 2.14.0). The percentage of Ki67-positive stained area was quantified by applying thresholding to five randomly selected fields per tissue and normalizing it against the corresponding haematoxylin-stained area.

***In vivo* external beam irradiation protocol (2 Gy x 6 only)**

Tumors were irradiated using the SmART+ Small Animal Radiotherapy System (Precision X-Ray, Inc., Madison, CT, USA). Mice were anaesthetised and placed in a supine position; the temperature of the irradiator chamber remained 35 °C through a heat lamp. Cone beam CT images of tumors were acquired for each mouse with a 2 mm aluminum filter and the mouse soft tissue high dose CT pre-set (40 kVp, 8 mA, 1x1 binning, 0.1 mm voxels; estimated dose to the centre-of-mass of the mouse of 12 cGy). The Monte Carlo irradiation treatment plan was created using SmART-ATP (Advanced Treatment Planning) Scientific Solutions software v2.0.20201216.

**Combination therapeutic study with 8 Gy x 1**

Female C57BL/6 mice were anaesthetized as previously described and inoculated with MTCQ1 cells at 1x10⁶ cells per mouse. Once tumors reached an average volume of 100 ± 34.3 mm³, mice were randomized using Randomice software into the following groups: (i) CT-only control, (ii) 8 Gy x 1 group, receiving a single fraction of 8 Gy irradiation, (iii) 8 Gy x 1 concurrent group, receiving a single fraction of 8 Gy irradiation alongside the anti-PD-L1 regimen starting on the day of irradiation, and (iv) 8 Gy x 1 sequential group, receiving a single fraction of 8 Gy irradiation followed by the anti-PD-L1 regimen starting one day post-irradiation. Anti-PD-L1 was administered intraperitoneally. All irradiations were performed under anaesthesia, as previously described. Mice were allowed to recover and were monitored for health and tumor growth. Survival was assessed and presented as a Kaplan-Meier curve.

**SUPPLEMENTARY FIGURES**


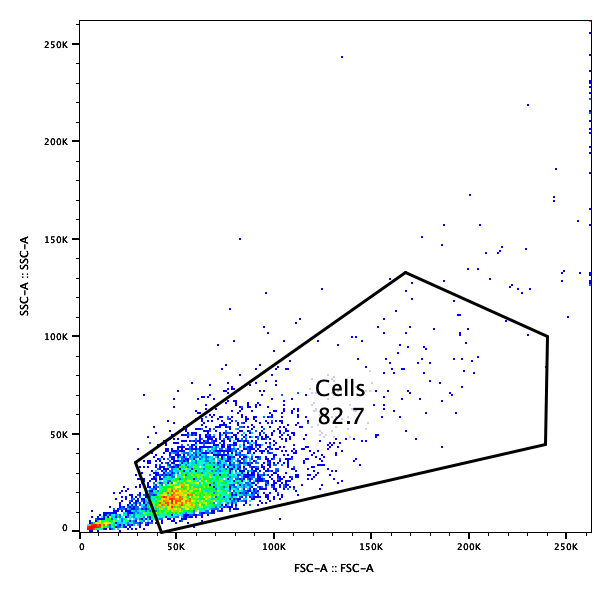

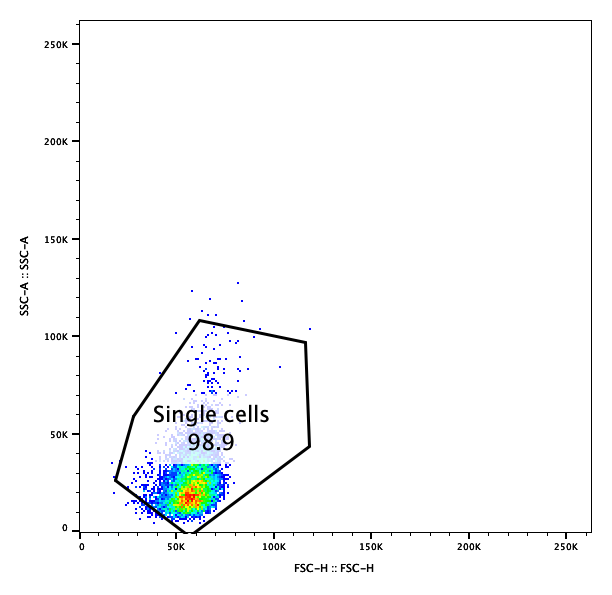

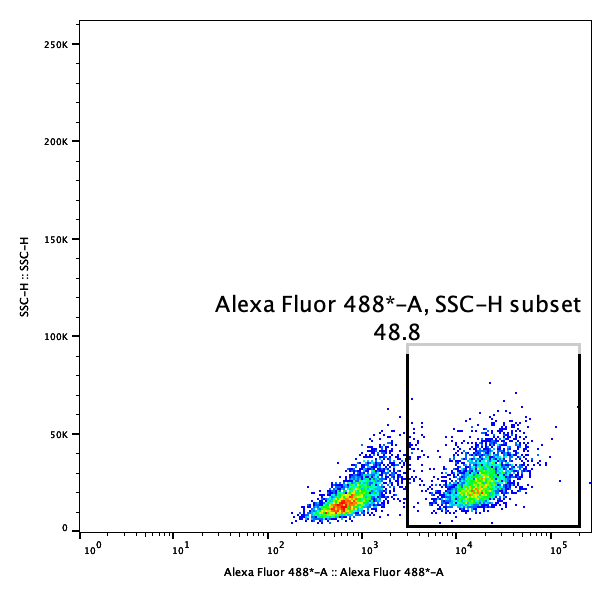

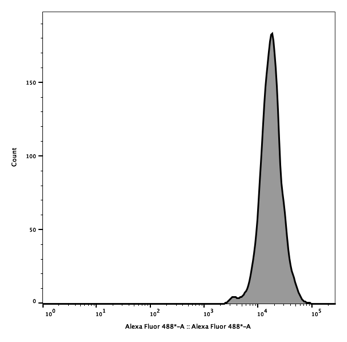


FSC-A

FSC-H

Alexa Fluor 488-A

Alexa Fluor 488-A

a

b

c

d

SSC-A

SSC-A

SSC-H

Count

Figure S1: Gating strategy for flow cytometry analysis of γH2AX detected using antibodies conjugated to Alexa Fluor 488. (a) Initial cell population selection based on size and granularity (forward scatter FSC-A vs. side scatter SSC-A). (b) Single cell isolation (FSC-H vs. SSC-A). (c) Alexa Fluor 488-positive subset identification. (d) Histogram showing the distribution of fluorescence intensity in the gated population.

**
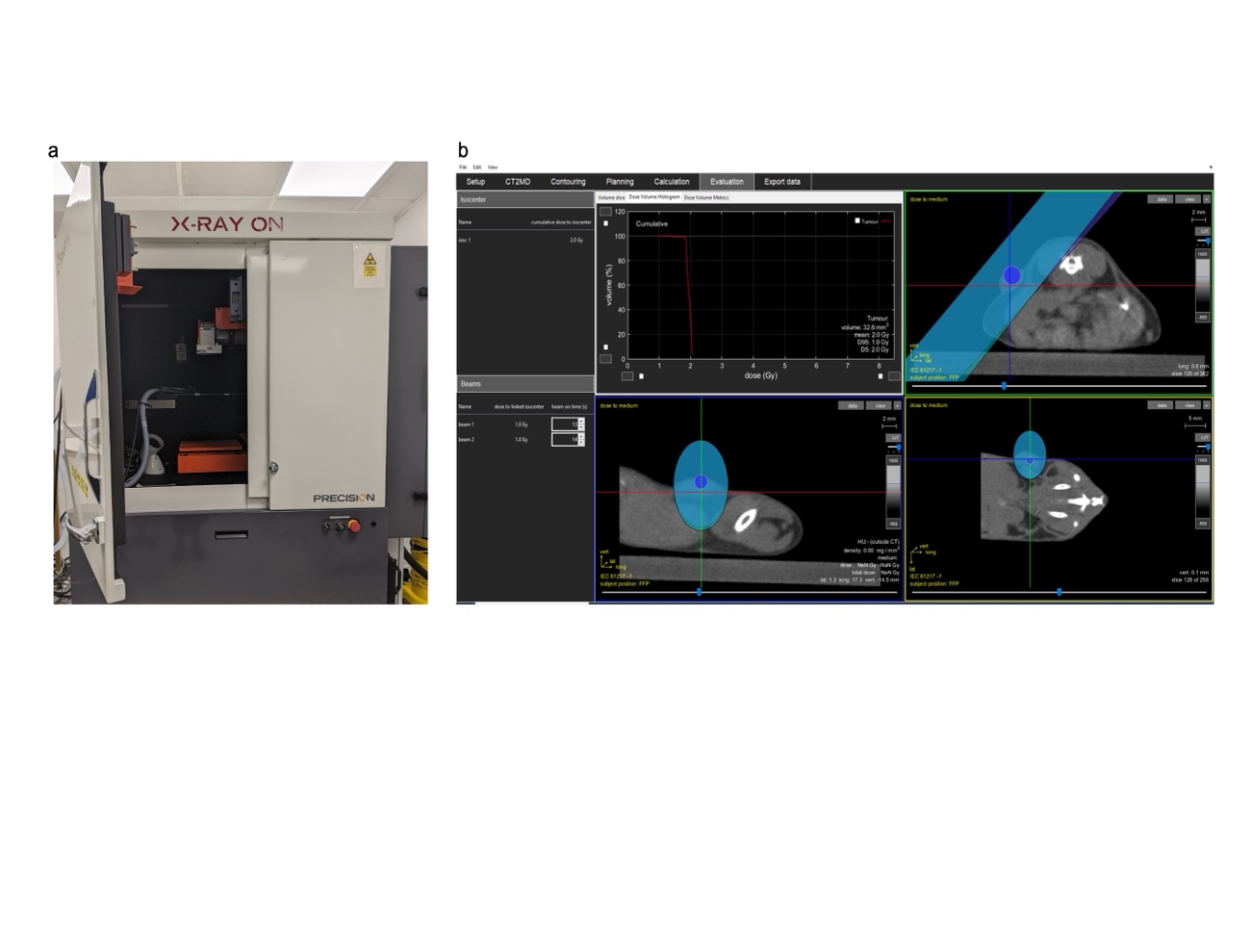
**

Figure S2: Treatment planning for radiotherapy. (a) SmART+ Small Animal Radiotherapy System, Precision X-Ray, Inc. (Madison, CT, USA). (b) Treatment planning after dose volume calculations using Monte Carlo simulations showing dose volume histogram (top left) and transverse/sagittal/coronal views of the X-ray beam (blue) pathway and isocenter (dark blue circle) passing through the tumor volume (outlined in red).


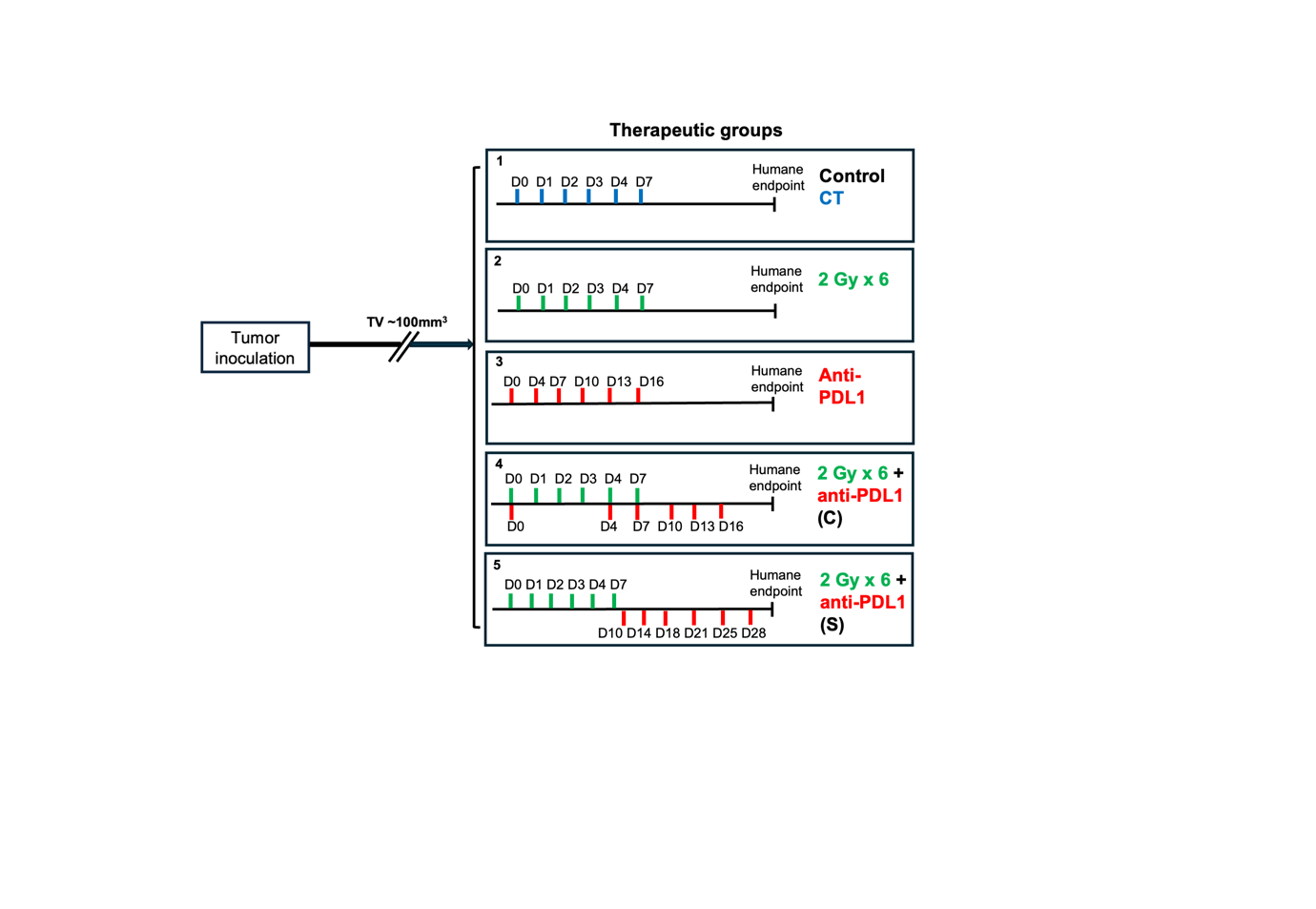


Figure S3: Once tumor volume (TV) reached approximately 100 ± 27.1 mm³, mice were randomized into five treatment groups, with interventions administered on specified days (D) as illustrated in the schematic.

1. Control (CT) Group: Mice received only CT scans (blue lines) without any therapeutic intervention.
2. Radiotherapy 2 Gy x 6 Group: Mice received six fractions of 2 Gy radiotherapy (green lines) excluding weekend.
3. Anti-PD-L1 Monotherapy Group: Mice received intraperitoneal injections of anti-PD-L1 (10 mg/kg) at designated time points (red lines).
4. Concurrent Combination Therapy Group: Mice received both 2 Gy x 6 radiotherapy (green lines) and anti-PD-L1 (red lines) administered concurrently, starting on the same day of radiation delivery.
5. Sequential Combination Therapy Group: Mice first received 2 Gy x 6 radiotherapy (green lines), followed by administration of anti-PD-L1 (red lines) starting three days after the final radiation dose.


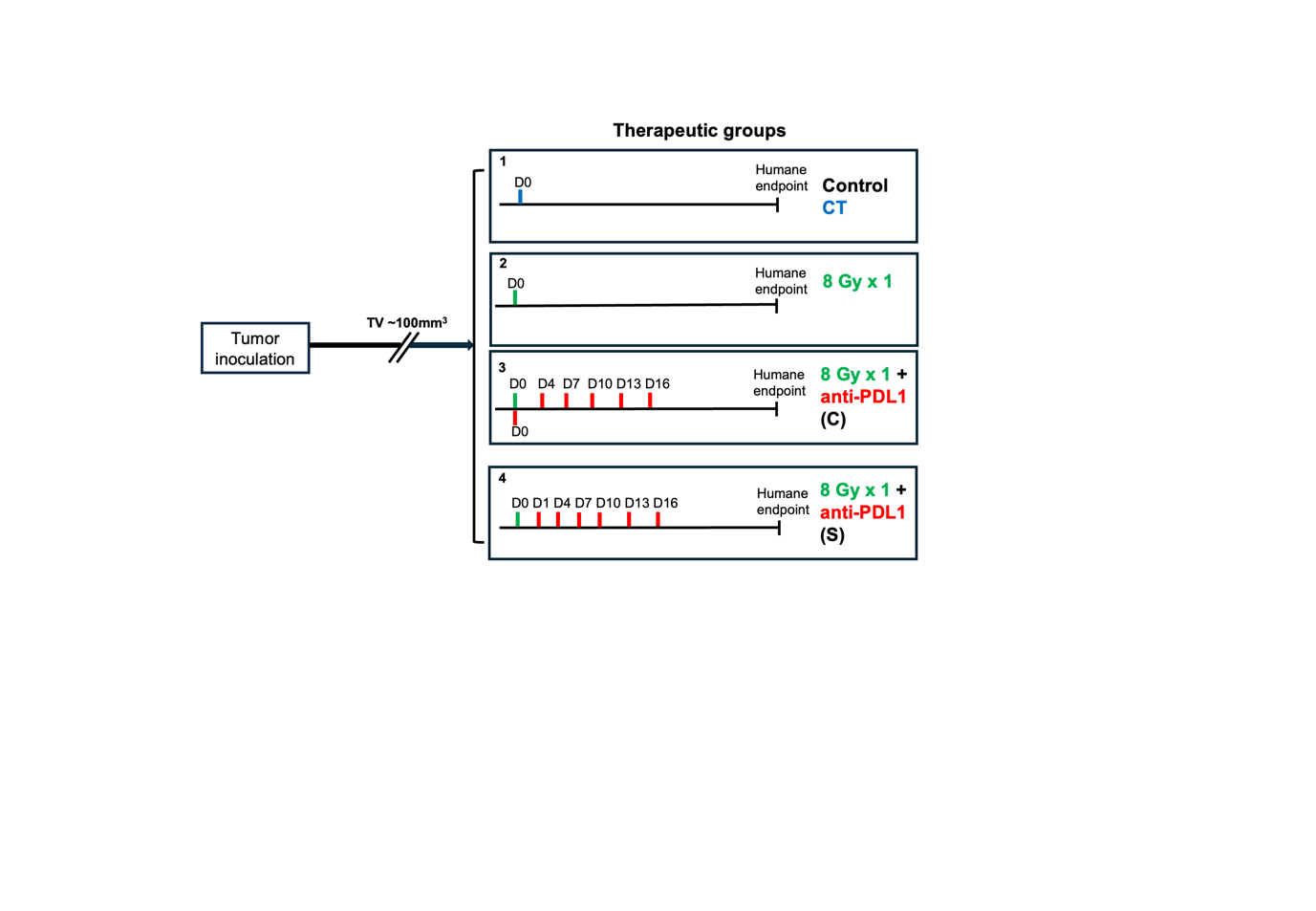


Figure S4: Once tumor volume (TV) reached approximately 100 ± 34.3 mm³, mice were randomized into four distinct treatment groups, with interventions administered on specific days as illustrated in the schematic:

1. **Control (CT) Group:** Mice underwent CT scans (blue line) without receiving any therapeutic intervention, serving as the baseline control.
2. **Radiotherapy 8 Gy x 1 Group:** Mice received single 8 Gy radiotherapy done (green line).
3. **Concurrent Combination Therapy Group:** Mice received both a single 8 Gy radiotherapy dose (green line) and anti-PD-L1 (red lines) concurrently, with anti-PD-L1 administration beginning on the same day as radiation delivery.
4. **Sequential Combination Therapy Group:** Mice first received a single 8 Gy radiotherapy dose (green line), followed by delayed administration of anti-PD-L1 (red lines), which commenced after day 1 post-radiation.


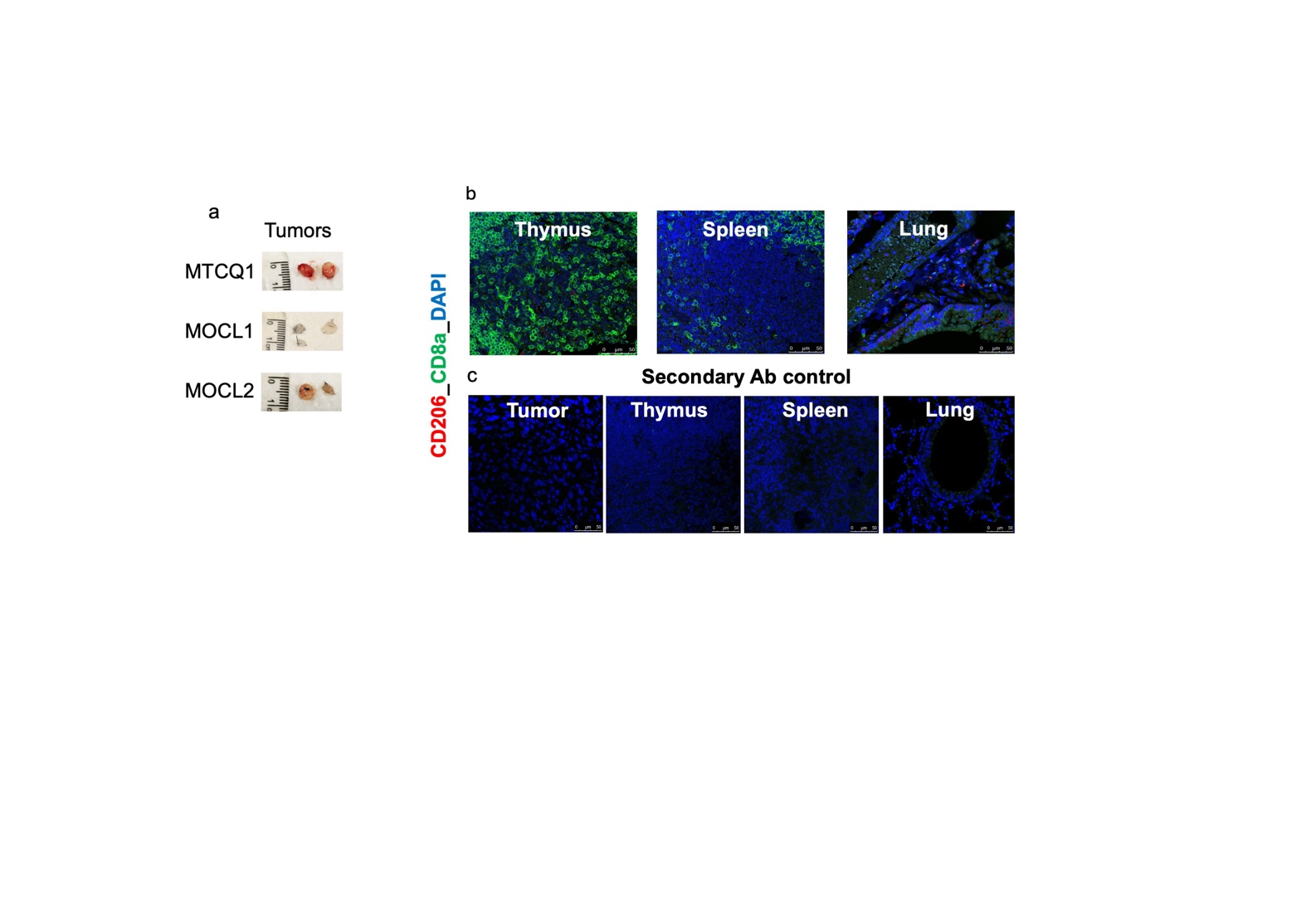


Figure S5: Establishing subcutaneous model and visualizing immune niche with confocal imaging. MTCQ1, MOCL1, and MOCL2 cells were injected with Matrigel in C57B/L6 female mice. (a) Macroscopic images of tumors collected on day 24 post inoculation. (b) Confocal images demonstrating the localization of CD206+ M2 macrophages (red), CD8a+ T cells (green), and nuclei counterstained with DAPI (blue) at the indicated organs of mice bearing MTCQ1 tumors (scale bar 50 µm). (c) Staining with secondary antibody only control for anti-CD8a and anti-CD206 in the indicated tissues.


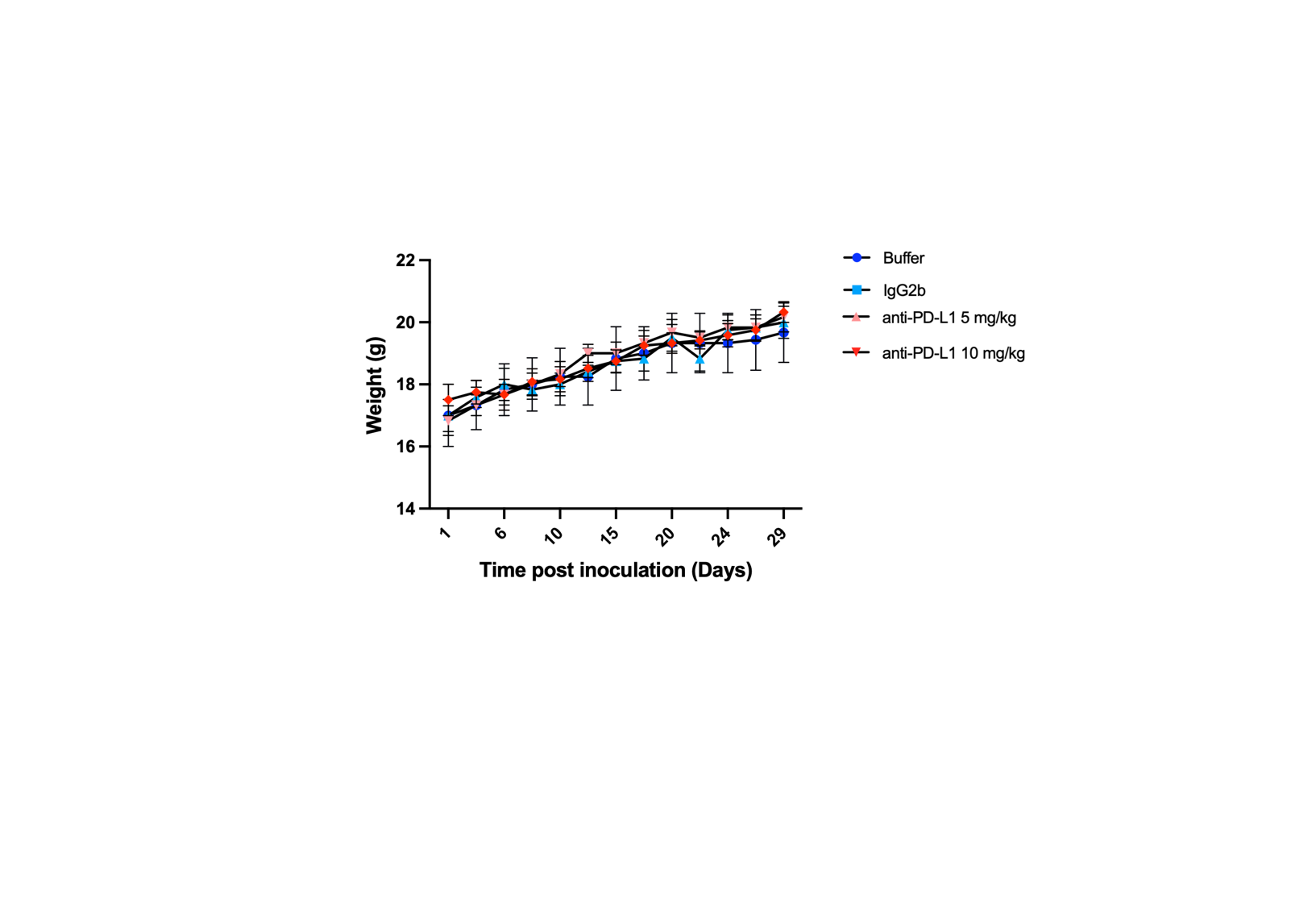


Figure S6: Weight of mice bearing MTCQ1 tumors treated with anti-PD-L1 compared to the controls. Data was analyzed by two-way ANOVA and is shown as average ± SD, with N=6 mice/group.


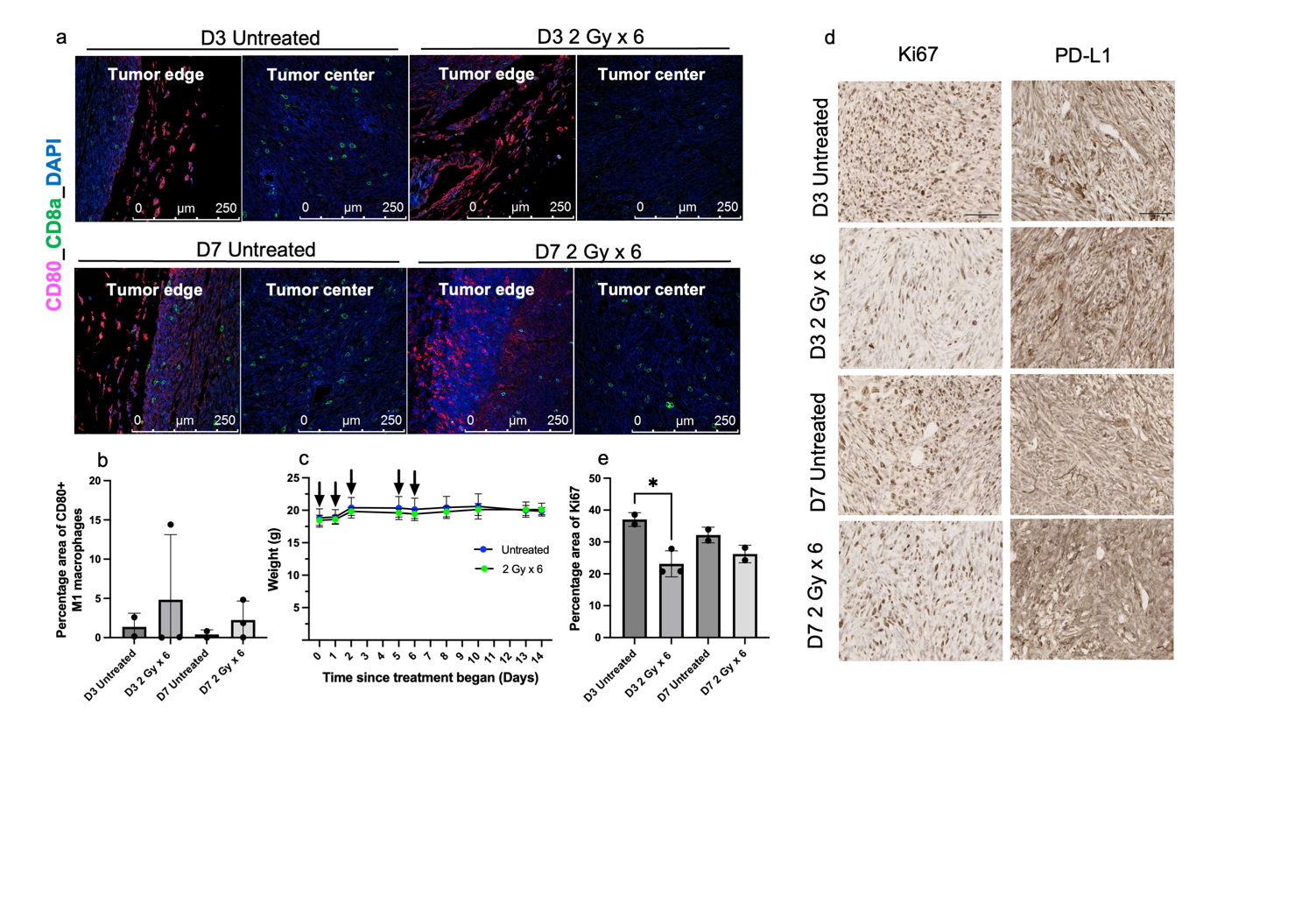


Figure S7: Time point study post 2 Gy x 6 irradiation of MTCQ1 tumors: (a) Confocal images of CD80+ M1 macrophages, and nuclei counterstained with DAPI (blue) (scale bar 250 µm). (b) Quantification of CD80+ M1 macrophage population in (a) and analyzed by one-way Anova. (c) Mouse weight analyzed by two-way ANOVA, with arrows (black) referring to the 2 Gy treatment timepoints. (d) PD-L1 and Ki67 staining in untreated and irradiated MTCQ1 tumors (scale bars 100 µm). (e) Quantification of Ki67 staining in (d) and analyzed by one-way ANOVA. Data is shown as average ± SD, N=3 mice/group. p*<0.05.


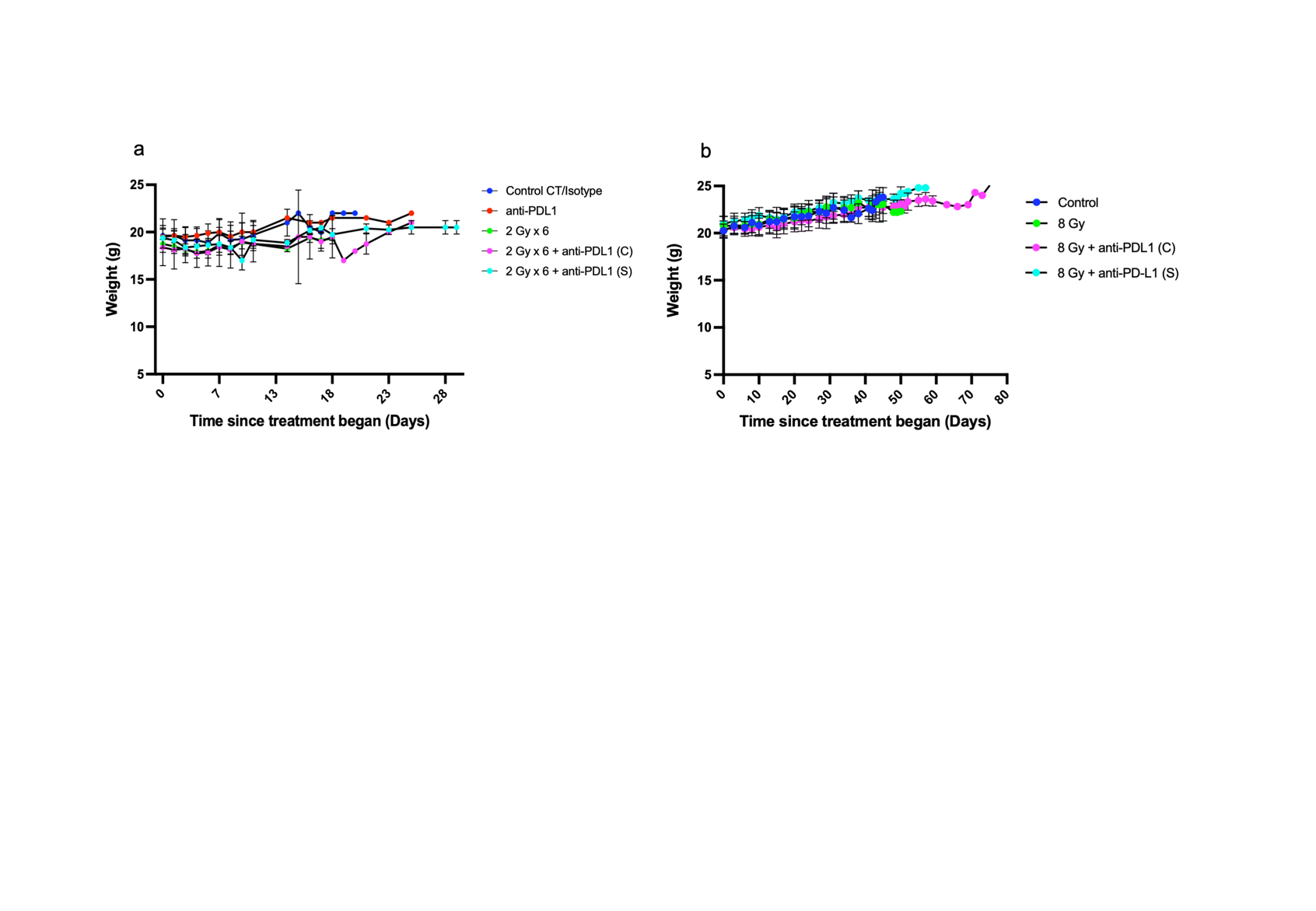


Figure S8: Mouse weights from the therapeutic studies using female C57BL/6 mice bearing MTCQ1 tumors treated with the indicated therapies (a and b). Data was analyzed by two-way ANOVA and represented as mean ± SD, N=8 mice per group.
